# Provider views on childhood obesity management in primary care settings: a mixed methods analysis

**DOI:** 10.1186/s12913-018-2870-y

**Published:** 2018-01-30

**Authors:** Kyung E. Rhee, Stephanie Kessl, Sarah Lindback, Marshall Littman, Robert E. El-Kareh

**Affiliations:** 1 Department of Pediatrics, University of California, San Diego, 9500 Gilman Drive, MC 0874, La Jolla, CA 92093 USA; 2 Children’s Primary Care Medical Group, San Diego, CA USA; 3 University of California, San Diego, Departments of Biomedical Informatics and Medicine, La Jolla, CA USA

**Keywords:** Childhood obesity, Primary care, Weight management, Provider behaviors, Chronic care model, Collaborative care model

## Abstract

**Background:**

Pediatric providers are key players in the treatment of childhood obesity, yet rates of obesity management in the primary care setting are low. The goal of this study was to examine the views of pediatric providers on conducting obesity management in the primary care setting, and identify potential resources and care models that could facilitate delivery of this care.

**Methods:**

A mixed methods approach was utilized. Four focus groups were conducted with providers from a large pediatric network in San Diego County. Based on a priori and emerging themes, a questionnaire was developed and administered to the larger group of providers in this network.

**Results:**

Barriers to conducting obesity management fell into four categories: provider-level/individual (e.g., lack of knowledge and confidence), practice-based/systems-level (e.g., lack of time and resources), parent-level (e.g., poor motivation and follow-up), and environmental (e.g., lack of access to resources). Solutions centered around implementing a team approach to care (with case managers and health coaches) and electronic medical record changes to include best practice guidelines, increased ease of documentation, and delivery of standardized handouts/resources. Survey results revealed only 23.8% of providers wanted to conduct behavioral management of obesity. The most requested support was the introduction of a health educator in the office to deliver a brief behavioral intervention.

**Conclusion:**

While providers recognize the importance of addressing weight during a well-child visit, they do not want to conduct obesity management on their own. Future efforts to improve health outcomes for pediatric obesity should consider implementing a collaborative care approach.

## Background

Primary care providers (PCPs) have been identified as key players in the treatment and prevention of obesity by several national groups [1–3]. The American Academy of Pediatrics Committee on Nutrition, [1] the Expert Committee Recommendations (ECR) regarding the prevention, assessment, and treatment of child and adolescent overweight and obesity, [2] and the National Initiative for Children’s Healthcare Quality [3] recommend the routine use of Body Mass Index (BMI) growth charts and the discussion of healthy lifestyle habits during *all* health supervision visits. Subsequently, the Healthcare Effectiveness Data and Information Set (HEDIS) 2009 incorporated BMI assessment and nutrition and physical activity (PA) counseling as quality measures that may be used by insurers to determine reimbursement in pay-for-performance programs, particularly if they have prevented the development of later disease [4]. The Affordable Care Act (ACA) also aims to improve obesity-related prevention and treatment coverage for children by improving access to health care services that support healthy weight [5].

Despite these recommendations, several studies document that rates of overweight/obesity (OW/OB) (overweight = BMI ≥ 85th and <95th percentile for sex and age based on the Centers for Disease Control and Prevention growth charts; obesity = BMI ≥ 95th percentile for sex and age based on the Centers for Disease Control and Prevention growth charts) [6] identification in pediatric primary care clinics are low, [7, 8] with less than 30% of overweight children being identified by their provider, [9–11] and less than 10% receiving an ICD-9 or − 10 diagnosis of overweight on billing forms [12, 13]. Even when weight assessments have been performed, PCPs often do not take the next step to engage patients or their caregivers in weight management discussions [14]. A recent national study demonstrated that there were no changes in diet/nutrition and exercise counseling after the release of the Expert Committee Recommendations in 2007, and that counseling rates decreased in high-risk populations [15].

There may be several reasons contributing to this low rate of weight management in the primary care clinics. First, PCPs often report low confidence in their ability to counsel and treat obesity as well as a lack of time and resources [16, 17] Specifically, PCPs report low proficiency in behavioral management skills and parent counseling techniques. Improving knowledge and skills to provide effective counseling during a busy outpatient clinic visit has been identified as a top priority by pediatric providers [18]. However, the strongest predictor of weight management behaviors among providers appear to be the availability of resources, specifically time, staff support, BMI calculation tools, and community resources [8]. These findings suggest that knowledge of behavioral management skills and counseling techniques among providers may not be enough to promote these discussions.

In addition, recent studies have shown that higher intensity (> 25 contact hours), multi-component behavioral interventions appear to be most effective at improving weight status [19, 20]. However, the U.S. Preventive Services Task Force (USPSTF) noted that moderate intensity counseling may not be deliverable by PCPs within the confines and structure of the current well-child visit, and that these children should be referred to intensive counseling/behavioral programs to assist with weight loss [21]. Unfortunately, intensive weight-loss programs often exist in tertiary care academic settings, and these programs can be difficult for families to access due to long wait lists or long distances to travel. Given the low proficiency of providers and difficulty of providing intensive counseling in the current primary care structure, other care models may need to be developed in order to comply with national recommendations to deliver this type of treatment in the primary care setting.

The goal of this study was to examine the views of pediatric providers on conducting obesity management in the primary care setting, and identify potential resources, structures, and care models that could facilitate the delivery of more effective obesity management in these clinics. Using a mixed methods approach, we first conducted focus groups among providers in a large pediatric network in San Diego County. A follow-up survey was then developed based on the focus group discussions and distributed among the larger group of pediatric providers in the area to assess their views on obesity management and the suggested solutions.

## Methods

### Focus group

We conducted four focus group discussions in December 2013 among pediatric providers in the Children’s Primary Care Medical Group (CPCMG), a large pediatric network in San Diego County. CPCMG has 20 offices in San Diego County and southern Riverside County with over 110 physicians, servicing a wide range of racial and socioeconomic regions. In conjunction with the CPCMG leadership, four sites in four demographically different regions of San Diego were identified to participate in the focus group to ensure that a wide range of practice experiences were represented. These areas included Poway, La Jolla, Escondido, and Chula Vista, and consisted of 26.1%–38.9% OW/OB children, 15.7%–58.2% Hispanic, and 5.9%–23.2% of families living below the federal poverty level. All pediatric providers (physicians and nurse practitioners) at each site were invited to participate in the focus group which occurred in their office during the lunch hour. Twenty-two providers (physicians and nurse practitioners) participated. Lunch was provided, but no other means of compensation was offered. The study was approved by the Institutional Review Board at the University of California, San Diego.

Focus group discussions were conducted in English and consisted of 2 to 10 providers. While themes started to converge by the second and third focus group, four focus groups were conducted (one at each site) to ensure that all demographic locations would be represented. Before the start of each discussion, providers completed the informed consent process and a short (10-item) questionnaire assessing their age, demographic characteristics, medical training, and training in obesity management. Focus group discussions were conducted by a researcher trained in qualitative methods and conducting focus groups (KR). A facilitator was present to take notes and assist with follow-up questions. A semi-structured interview guide was developed by the research team and based on researcher expertise, experience of the CPCMG leadership, and empirical literature. Topics included: current attitudes and behaviors towards weight management in the office; barriers to addressing weight in the office setting; and factors or solutions that would increase providers’ abilities to manage weight in the office (e.g., resources, staffing, electronic tools). The focus group leader followed this guide and allowed participants to openly express their opinions. Since focus group members worked together in the office, numbers were not assigned to protect anonymity. However, it was reiterated to the group that all responses would remain confidential and anonymous during the coding and reporting process. Focus group discussions were 45 min in length. Discussions were audio-taped, and the tapes transcribed verbatim for analysis. Transcriptions were entered into qualitative software (Atlas.ti Version 7.5.11 (2016, Scientific Software Development GmbH, Berlin)) for analysis.

### Analysis

Qualitative methods were used to analyze the focus group discussions [22]. Transcripts were independently coded by two authors (KR and SK). First, one investigator read all transcribed focus groups and applied the principles of microanalysis, [23] an in-depth analysis of the text to generate initial themes and create a preliminary coding scheme. The second investigator (SK) then attempted to apply the initial coding scheme to each transcript. The investigators met to refine the coding scheme and discuss new emergent themes using the constant comparison method [23]. Consensus was reached on the definition and application of each theme. Codes were associated with segments of dialogue based on a priori themes (i.e., questions asked in the focus group) or emergent themes (i.e., central ideas from the data). Different codes could be applied to the same segments of the transcript. Atlas.ti Version 7.5.11 (2016, Scientific Software Development GmbH, Berlin) was used to organize codes and their subcategories. SAS v9.4 (SAS Institute Inc., Cary, NC) was used to obtain descriptive statistics of the sample.

### Survey

Based on the results of the focus group discussions, a 30-item questionnaire was developed to assess the views of the overall group of CPCMG providers on obesity management. The questions were developed to assess current practices in obesity management, identify opportunities to improve practice, and understand the systems used in managing childhood obesity. All pediatric providers in the CPCMG system (*n* = 110) (including those who had participated in the focus group) were sent an e-mail invitation from the CPCMG leadership to participate in the online survey; 42 (38.2%) providers responded. Data from the questionnaire were downloaded into excel and used for analysis.

### Questionnaire items and analysis

Providers were asked on a 4-point Likert-type scale how important it was to address weight with their OW/OB patients (1 = not important, 4 = very important). They were also asked to indicate what they felt their role should be in the management of pediatric obesity and check all the responses that were appropriate. Providers were then asked to report how effective they were in the behavioral management of obesity (4-point Likert-type scale, 1 = not very effective, 4 = very effective). Based on the focus group discussion, a list of obesity management choices were offered and providers were asked to rank their choices from most desirable (1) to least desirable (7). Choices ranged from group visits for families during regular office hours to a health educator or nurse who would engage in behavioral weight loss management in the office (see Table 3 for full list of options). Providers were then asked about specific electronic medical record (EMR) changes that they would like to see to help them with obesity management in their clinic setting. Providers were allowed to choose as many options as they deemed suitable. Providers were also asked about their knowledge of community resources and how they would like to administer surveys. Demographic characteristics (sex, years in practice), type of medical training, and prior obesity training (CME courses) were ascertained at the end. Descriptive analysis, including means and frequencies, was conducted using SAS v9.4 (SAS Institute Inc., Cary, NC).

## Results

### Focus group

A total of 22 providers participated in the focus groups. The majority (68.2%) were female, 81.8% Caucasian, and 77.3% physicians. One-third (36.3%) of the group had been practicing for 10 years or less, while 40.9% had been practicing for 11–20 years. Half of the group (50%) had never participated in childhood obesity training in the past, while 31.8% had participated in 1–5 obesity-related CME courses.

The majority of barriers fell into four categories: provider-level/personal barriers, practice-based/systems barriers, parent-level barriers, and environmental barriers (Table 1). Among provider-level barriers, the most commonly reported issues were lack of knowledge and confidence around obesity management, particularly around effective means of communication. The majority of providers were not comfortable with offering weight management advice and delivering it in a meaningful and effective manner. Many providers reported being nervous about addressing this topic with their patients and parents for fear of offending them.

**Table 1:** Barriers associated with obesity management in the primary care setting

Barriers	Examples of comments
Provider-level/ Personal: Lack of knowledge and confidence, poor communication skills	• “I feel nervous, like I’m in a very dangerous place, because I don’t want to mess anybody up and make it so they’re less happy. They’re already unhappy with themselves…”
	• “I worry about using the words overweight and obese.”
	• “So it stresses me out a little bit because some kid’s BMI may be over 85th percentile but they are an athlete or are otherwise healthy or the family is otherwise thin and they are going to grow out of it.”
	• “It takes a few visits so that they trust you and…you are not judging them.”
	• “The bigger problem with everything is actually knowing where – what to do for these folks.”
Practice-based/ systems level: Lack of time, poor training, lack of resources	• “I would say there is a serious time management issue because there is a lot of material that we are supposed to be covering during the well-child check and although yes I know how to code for that extra visit at your well-child check, you don’t magically make time appear out of thin air… Getting the parents to buy in and getting the kid to buy in - that is an issue that takes time.”
	• “We don’t want to go in and make it a big thing and make a 10 min visit into 30 min.”
	• “So we need some more training… What works?... Do those scare tactics work? Has anyone studied it?”
	• “I’ve had [motivational interviewing training] a little bit, but I’m not a professional in that respect, and I’d love to have someone who knows how to word it better than I do add it to our well-child visits.”
	• “Even within [our] community there is a lot of confusion among the subspecialists as to who is dealing with what. What is the difference between [A] clinic and [B] clinic?”
Parent-level: Poor motivation, readiness, follow-up, and recognition of their child’s weight issues	• “If they don’t think they’re overweight it’s really hard to get them to do stuff.”
	• “I find it successful only if the patient has identified the problem and put it as their concern.”
	• “If they are just a little bit obese, I will give them a 3 month period to diet and exercise, then come back and check everything… very rarely do they ever come back for that 3 month visit.”
	• “I always tell them let’s come back… and they never come back.”
Environmental: Lack of access to services and lack of transportation	• “Some of the problem I get is the access. If it’s like after school or… at night so the parents could go, but then the parents can’t go because they do not have day care you know, a babysitter for the other kids, or they have to work at night. They are doing the two job thing.”
	• “Parents don’t like to travel, and they don’t have cars. Some people have to take the bus.”
	• “If you bring it up for the parents they say, ‘It sounds great but I cannot drive there’.”

The most common practice-based or systems level barriers included lack of time, poor training, and lack of resources in the office and community (Table 1). Providers expressed interest in learning more about what strategies to use to help children lose weight and how to use techniques like motivational interviewing in the office setting. However, lack of time limited their ability to engage in these discussions. Providers also expressed uncertainty regarding community resources, existing treatments, and where to refer their patients. Providers reported that they “*didn’t know where else to send [their patients]*,” and expressed a desire to have a “*simple way of knowing…where or even what’s available [in the community].”*

Lack of parent motivation and readiness to address obesity was the third most commonly reported barrier. Providers felt that motivating parents to engage in treatment was difficult, particularly if parents did not recognize that their child was overweight (Table 1). Finally, providers identified several environmental-level issues related to access, specifically a lack of treatment options for their patients and an inability or unwillingness to travel among parents in order to participate in treatment.

When discussing possible solutions, providers’ responses centered around 2 major themes: systems-level changes in the office and specific work-flow changes, many of which involved the EMR (Table 2). When discussing systems-level changes, many providers requested additional support in the form of a clinical educator or nutritionist to help deliver effective obesity management strategies in the office. Providers also thought that if this care could be provided in the office, it would be beneficial. Of note, providers were open to addressing obesity in the primary care setting if there was a team approach. However, if education or counseling could not be done in the office, providers also identified the need for a referral coordinator to help manage these patients.

**Table 2:** Potential Solutions for obesity management in the primary care setting

Solutions	Examples of comments
Systems level: Ancillary support in the office, obesity champion, obesity management guidelines, team approach, more training	• “I think making it somebody who is very knowledgeable that’s not necessarily a physician would make more sense. And figure out the compensation model and if there was enough money.”
	• “If there was a clinical educator option that would be so awesome because… you could say [to the family] that I think this is really important, it’s a big deal… I’m gonna set you up.”
	• “So doing it closer to home would make more sense, in terms of having the follow through, and then the loop back… This is their home office and they feel comfortable coming here.”
	• “I’m willing to give [obesity management] the energy if you are willing to actually absorb some of it by being this team with me, but not if I’m in it by myself.”
	• “Maybe a conjunction - an MD and a nutritionist would be really good.”
	• “I think it would be great to… think of… someone who is in charge of it, like a champion.”
	• “I mean… something like an algorithm that could be given to [x person] so that we just had to… (I know we kind of passed the buck here), but… do a referral for obesity, and it goes to [this person]. She looks down the algorithm to where they go and she then sends them over to where the best resource is, opposed to us having to think about it on every single patient, you know, because she is the referral coordinator.”
Work-flow changes: Develop best practice guidelines, ease of documentation, standardize resources and handouts, gather family-level information	• “[Get] a protocol for when we all have a kid in the office on a well-check…We should have a button saying return in a month or return in two weeks or whatever the return time is. That is when you bring them back and drop down to your obesity smart set with your labs because you have to.”
	• “In our checkup one of the things that gets billed for… is what the child’s BMI is… I sometimes won’t label [the child as]… obese, I will say BMI over 95th percentile, and I put it in their problem list because parents get very touchy if they see [obese] in their problem list, and I do not know if that is ok for monitoring…I don’t know what we should put on the problem list.”
	• “More help with the documentation part of it and making it easier.”
	• “An obesity follow-up note. It might be nice to have a [smart form or obesity template] like that.”
	• “The [handouts] need to be in EPIC for our use and also if there’s any other websites.”
	• “What if there was like a separate survey that we could give to families…so if they came in and we noticed they were heavy or obese… we would give them a survey that they would maybe bring back to their next [visit]. Like have you tried anything? Do you see this as a problem? Tell me about the foods you eat in your house… Kind of getting a feel for it so they start looking at what we’re looking at, and they could come back with that information, and so it’s something that we could have as an after visit summary thing [in EPIC]. Then we can say, you know what, why don’t you do this?… That way when you come in we’re going to have a really good place to jump off from and we’ll know more.

Providers also expressed an interest in having a clear obesity management guideline or best practice guideline within the EMR to help them streamline their care and ensure consistency between providers. In particular, providers wanted guidelines on what labs to order, what referrals to make, and when to bring a patient back for a weight check or additional counseling (Table 2). They were also interested in having evidence-based handouts, links to online resources, and a directory of active community programs and resources. Efforts to make documentation easier (e.g., in the form of smart sets, smart forms, obesity management templates, drop-down menus, and “dot phrases” or text expanders) were also important to providers. Given the intensity of the conversation needed for obesity management, these forms would help to standardize the interaction, but also decrease the documentation burden of an already busy encounter.

### Survey

**Fig. 1:**
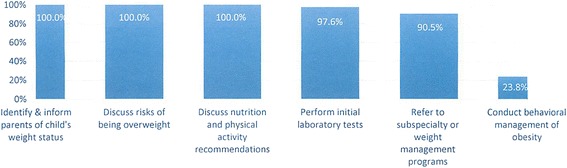
Pediatric Providers’ Views on Their Role in the Management of Childhood Obesity? A total of 42 (38.2%) of providers responded to the survey. Providers were allowed to choose as many as options as they thought were appropriate

Based on the focus group results, a 30-item questionnaire was developed to gather the views of the entire network of providers. Of the provider survey respondents (*n* = 42), 52.5% were female, 90% were physicians, and 72.5% were 11–20+ years post training. All of the providers thought it was either very important (83.3%) or important (16.7%) for them to address the issue of obesity with their patients. When asked what their role should be in obesity management, the majority indicated that they should inform parents of their child’s weight status, discuss nutrition and physical activity recommendations, conduct laboratory tests, and refer to subspecialty or other weight management services (Fig. 1). However, only 23.8% indicated that providers should be the ones to conduct the behavioral management of obesity. Furthermore, only 23.8% reported that they were “effective” or “very effective” at the behavioral management of obesity. Nevertheless, 90.5% reported that they were interested in learning more about this type of management.

**Fig. 2:**
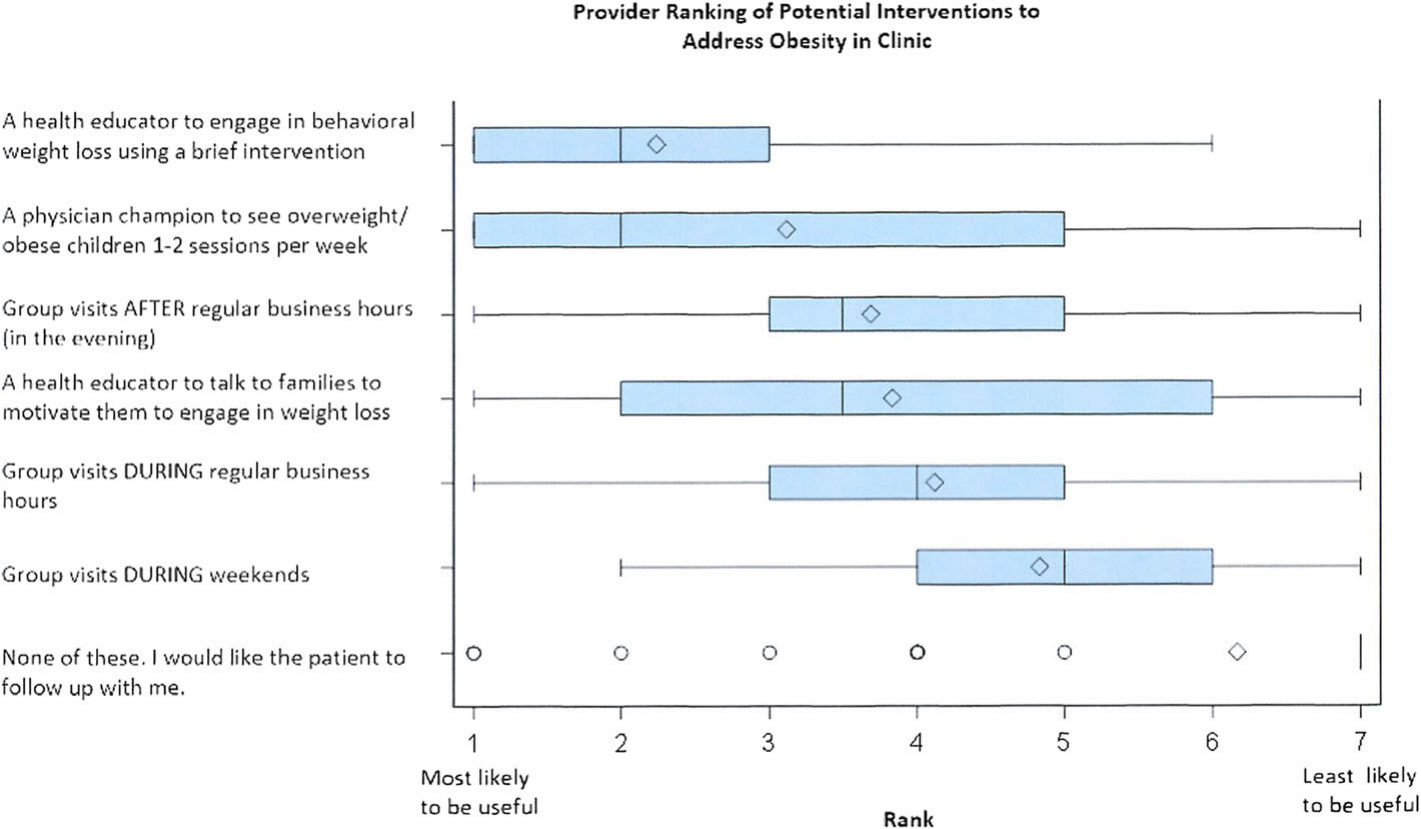
Provider Ranking of Potential Interventions to Address Obesity in Clinic. Providers were asked to rank several interventions they would like to see in their clinic. The line in the middle of the box represents the median; diamonds represent the mean. Edges of the box represent the 25th and 75th percentile interquartile range; whiskers represent the minimum and maximum observation

Additional questions were asked to determine what changes would help them streamline their ability to conduct obesity management in the clinic setting. The most highly ranked options were a trained health educator or physician champion who would engage in brief behavior change counseling and obesity management in the office (Fig. 2).

Finally, providers were asked to indicate how the EMR could help them better manage overweight children. Almost 2/3 indicated that they would like a Best Practice Advisory (BPA) to appear if the child had a BMI ≥ 85th percentile. Among those who said yes, 80.8% preferred a smart set or algorithm that they could “opt” to use to help them with subsequent management; less than 20% preferred to have a “hard stop” that forced them to order labs, make referrals, and provide handouts.

There were several options within this smart set that providers wanted (Table 3). First, providers suggested there should be a mechanism in the chart to help them recognize that the child’s BMI was in the OW/OB range, preferably highlighted in the progress note. They also identified a number of tools they thought would be helpful, such as a guide for what labs to order, appropriate nutrition and physical activity assessments, and a list of appropriate diagnosis codes. Templated progress notes would help to streamline the documentation process, and standardized handouts and referral links were requested. Providers were open to the idea of administering a survey to families to help gather more information regarding obesity-related behaviors. They indicated that they would like the medical assistant (MA) to provide this survey to the family to complete in the office (70%), or they would give the family the survey to complete at home and have them return to a follow-up visit to discuss their responses (67.5%). Of note, 90.2% of providers indicated that they did not have adequate knowledge of the available resources in the community to help their families with weight loss, and many preferred a link in the EMR to help them learn about these resources.

**Table 3:** Preferred Options for Obesity Management in the Electronic Medical Record (EMR)

		% of providers
Options to help providers identify the child’s weight status	- Highlight BMI in the progress note if child is overweight/obese	73.0%
	- Implement an alert system	48.7%
	- Add it to the title bar	27.0%
	- Make it a “hard stop” if it is not on the problem list	10.8%
Options in an obesity smart set	- What labs to order	94.1%
	- Nutrition and physical activity assessments	92.5%
	- Diagnosis codes	91.2%
	- Templated progress note	85.3%
	- Standardized handouts	85.3%
	- Information regarding community resources	82.6%
	- Referral links	79.4%
	- Readiness to change questions	76.5%
	- Behavioral management toolkit	61.8%
Handouts or resources available in the EMR	- Portion size recommendations for each age group	97.4%
	- Physical activity suggestions	92.3%
	- Grocery list of suggested healthy food items	89.7%
	- Food diary templates	84.6%
	- Links to appropriate websites	84.6%
	- List of effective behavioral strategies	79.5%
	- List of local community resources for nutrition, physical activity, and weight loss	66.7%
	- Reward charts	56.4%
Methods of administering questionnaires in the office	- Medical assistant provides questionnaire to parent to complete during the visit	70%
	- Physician gives family questionnaire to complete and return with the weight-check follow-up visit	67.5%
	- Provide questionnaire via ‘MyChart’ (EMR web portal)	50%
Assistance with follow-up appointments	- A tool to help identify and send out follow-up reminders	67.5%
	- A separate smart set for follow-up weight check appointments	83.9%
Method of learning about community resources for obesity management	- Link in EMR	87.8%
	- E-mail	53.7%
	- Personal meeting with community representative	41.5%
	- Webinar	29.3%

## Discussion

The purpose of this focus group and survey was to explore the challenges and possible solutions for conducting more effective pediatric obesity management in the primary care setting. The physicians and nurse practitioners in San Diego County reiterated many of the commonly recognized challenges, namely the lack of time, poor communication skills, and limited resources to effectively conduct this management within the structure of the current well-child visit. These sentiments echo those from previous reports [16, 17] and highlight the continued difficulty providers have with conducting obesity management in the office setting. Providers also commented on the need to increase families’ motivation to engage in treatment and identify trusted local referral resources that families could easily access. This issue of access has not been frequently highlighted in previous studies and underscores the need to develop effective programs that can be implemented in the primary care setting or other community settings that families from underserved communities can easily access.

Given the constraints of conducting obesity management during a well-child visit, several providers offered solutions that mirror those defined in the chronic care model of treatment [24]. In a chronic care model, providers and health systems work together to improve management of chronic conditions. This is accomplished by implementing decision support tools and clinical information systems to assist providers, providing self-management support and case management to help patients successfully navigate the health care system, providing support for patients to make health behavior changes, and linking the patient to different components of the health care system and community resources [25]. Providers in this study identified many decision support and EMR modifications that they perceived would help them streamline their management of obese patients, including templated progress notes, standardized handouts, links to nutrition and physical activity assessments, and smart sets for labs, diagnosis codes, and community and subspecialty referrals.

However, focusing primarily on EMR and decision support changes may only provide a modest impact on health outcome measures, with a greater impact on process measures [26]. Changes to the care team and the behavioral management of obesity should be made in concert with EMR changes to potentially make a larger impact. Additional ideas generated by this group included a team approach to childhood obesity management where a health educator provides behavioral strategies to families and works to increase motivation and engagement in the weight management process. Providers felt that this kind of support would address two important barriers: (1) the lack of time they had to provide more thorough obesity management, and (2) the lack of confidence in their communication and motivational interviewing skills.

Given these responses, the collaborative care model may be a compelling option for the delivery of weight management in the primary care office. This model utilizes a team approach and typically includes a care manager or health coach who supports patient self-management behaviors by delivering structured management plans and brief behavioral interventions [27]. They would also schedule frequent follow-ups, provide care coordination, and increase patient/parent awareness of the resources in the community that will support healthy eating and activity behaviors. This model provides the support patients need to make behavior changes while also allowing each member of the care team to provide the best quality of care within his or her scope of practice. This model would then lead to a more efficient and effective use of skills and resources. With the delivery of an effective but brief behavioral intervention for weight loss, application of the collaborative care model may result in improved health outcomes for overweight children, as well as increased access for families, and greater satisfaction with obesity care in the primary care setting.

Despite the usefulness of the findings of the focus group discussions and survey, there were some limitations. Primarily, the evaluations were conducted within one large pediatric primary care group in San Diego County. The responses and suggestions of this group may have reflected the nuances of practicing in their health network and may not generalize to other populations. Of note, CPCMG is clinically integrated with the local children’s hospital where subspecialists can assist with recommendations regarding lab ordering, and clinical course and lab results often guide referral decisions. Despite this support, providers still expressed frustration with obesity management and many suggested implementing components of the chronic care model and collaborative care model of patient management. Another limitation to this study is the low response rate to the survey. Views of providers who were less enthusiastic about obesity management were likely not represented in these results. Increasing engagement in this group of providers may be difficult, which lends further support to the development of a collaborative care model and implementation of a care manager or health coach to help manage OW/OB children.

## Conclusion

The results of this mixed methods analysis reveal that while pediatric providers believe childhood obesity is an important issue to address, they cannot provide effective weight management on their own. They suggested implementing a team approach where a care manager or health coach supports patient/parent self-management behaviors, delivers brief behavioral interventions, and provides case management. Identifying effective but brief behavioral weight control interventions (such as a guided self-help treatment program that supports patients as they make tailored behavior changes that fit their lifestyle and readiness to change level [28]) will be key to the success of this model. Providing this care within or close to the primary care setting will also be important to improve access for patients. Future efforts to improve health outcomes for pediatric obesity should incorporate a comprehensive system of change that takes advantage of the strengths and skills of each team member and also provides effective behavior change support and access for families.

## Abbreviations

ACA: The affordable care act
BMI: Body mass index
BPA: Best practice advisory
CPCMG: Children’s Primary Care Medical Group
ECR: Expert committee recommendations
EMR: Electronic medical record
HEDIS: Healthcare effectiveness data and information set
MA: Medical assistant
OW/OB: Overweight/obesity
PA: Physical activity
PCPs: Primary care providers
USPSTF: U.S. Preventive Services Task Force
